# Effects of Rutin and Hesperidin and their Al(III) and Cu(II) Complexes on *in Vitro* Plasma Coagulation Assays

**DOI:** 10.3390/molecules16021378

**Published:** 2011-02-07

**Authors:** Vesna Kuntić, Ivana Filipović, Zorica Vujić

**Affiliations:** 1 Faculty of Pharmacy, University of Belgrade, 11000 Belgrade, Vojvode Stepe 450, Serbia; 2 Haematological laboratory, Institute of Oncology and Radiology of Serbia, 11000 Belgrade, Pasterova 14, Serbia

**Keywords:** rutin, hesperidin, flavonoid-metal complexes, anticoagulation action, aPTT, PT, TT

## Abstract

Two flavonoids, rutin and hesperidin, were investigated *in vitro* for anticoagulant activity through coagulation tests: activated partial thromboplastin time (aPTT), prothrombin time (PT) and thrombin time (TT). Only an ethanolic solution of rutin at the concentration of 830 µM prolonged aPTT, while TT and PT were unaffected. In order to evaluate whether the prolongation of aPTT was due to the decrease of coagulation factors, the experiment with deficient plasma was performed, showing the effects on factors VIII and IX. Since pharmacological activity of flavonoids is believed to increase when they are coordinated with metal ions, complexes of these flavonoids with Al(III) and Cu(II) ions were also tested. The results showed that complexes significantly prolonged aPTT and had no effects on PT and TT. Assay with deficient plasma (plasma having the investigated factor at less then 1%) revealed that complexes could bind to the coagulation factors, what may lead to a non-specific inhibition and aPTT prolongation. An effort was made to correlate stability of complexes with their anticoagulant properties.

## 1. Introduction

Flavonoids are a ubiquitous group of polyphenolic substances which are present in most plants, concentrating in seeds, fruit skin or peel, bark and flowers. Numerous investigations of flavonoids in recent years described their beneficial pharmacological properties, such as antitumor, antibacterial, and antimutagenic activity, as well as cardiovascular protection [1,2,3,4]. The most important function of flavonoids, which underlies many of the above action in the body, is their antioxidant activity, *i.e.* ability to scavenge reactive oxygen and nitrogen species and radicals [5,6,7,8]. The antioxidant action of flavonoids is not only caused by direct reaction with free radicals, as flavonoids may also sequester metal ions by chelation and prevent the metal-mediated generation of free radicals and accordingly may protect the potential biological targets from oxidative stress [9,10,11]. Thus, by chelating metal ions responsible for the production of reactive oxygen species, flavonoids form complex compounds.

Flavonoid-metal complex compounds are the subject of our longtime research during which we investigated properties, composition, complex formation features, stability constants, as well as analytical appraisal of approximately 40 complexes of flavonoids from different flavonoid subclasses with a number of metal ions or metal groups [12,13,14,15,16,17]. The biological effects of flavonoid-metal complexes, confirmed by other authors in numerous studies, showed that complexes are more effective then free flavonoids, meaning that physiological properties of mature flavonoids are enhanced upon complexation with metal ions. The list of experimental data showing that the chelates are considerably more effective free radical scavengers than the free flavonoids is numerous. All these data are available in various previous publications [18,19,20,21,22,23,24,25,26].

However, flavonoid-metal chelates have not been investigated in *in vitro* human plasma coagulation assays. Furthermore, neither complexes nor flavonoids have been characterized for anticoagulant action, because, in general, flavonoids do not fulfil the structural characteristics typically associated with anticoagulant activity. Besides the already known effect of flavonoids to inhibit platelet adhesion, aggregation, and secretion in *ex vivo* assays [27,28], as well as the interleukin 1-induced expression of tissue factor on human monocytes [29], only two flavonoids have been tested for their *in vitro* activity on plasma coagulation. Guglielmone *et al* found that quercetin 3-acetyl-7,3’,4’-trisulphate (ATS) and quercetin 3,7,3’,4’-tetrasulphate (QTS) obtained from *Flaveria bidentis* showed significant prolongation of the activated partial thromboplastin time (aPTT), less for the prothrombin time (PT), and had no effect on the thrombin time (TT) at a concentration of 1 mM [30]. A possible explanation of the effects of both flavonoids could be due to the different degree of sulphation of these molecules. ATS, and especially QTS, present an electronegativity that is not usually found in other flavonoid derivatives; consequently, the structural requirements that associate them to the physiological glycosaminoglycans could be fulfilled. The data about anticoagulant properties of other flavonoids, as well as flavonoid-metal complexes are not available in literature.

Thus, the aim of this work was to investigate and compare the effects of flavonoid-metal chelates on *in vitro* plasma coagulation with effects of mature flavonoids. For this research, two flavonoids of different subclasses: rutin and hesperidin, as well as their complexes with Al(III) and Cu(II) ions were selected. Although this is our preliminary result, the effort was made to correlate the obtained effects with our previous knowledge about complexes [12,13,14,15,16,17].

**Figure 1:**
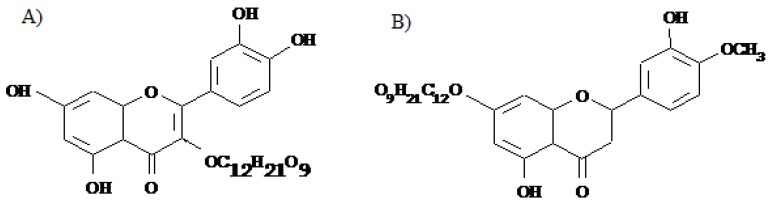
Structural formulae of rutin (A) and hesperidin (B).

Rutin (3, 3’,4’,5,7-pentahydroxyflavone-3-rhamnoglucoside) (hereinafter referred to as Rut, Figure 1A) is a flavonoid of the flavonol type, consisting of the flavonol quercetin and disaccharide rutinose (rhamnose and glucose). Rutin helps preventing hemorrhages and ruptures in the capillaries and connective tissue, and is therefore often used to treat chronic venous insufficiency [31].

Hesperidin (hesperetin 7-rhamnoglucoside) (hereinafter referred as Hesp, Figure 1B), belongs to the flavanone type flavonoids, predominantly occurring in *Citrus* species. Hesp is thought to reduce capillary permeability and to have anti-inflammatory action, hence it is used to shrink hemorrhoids, reduce varicose veins and battle viral infections [32].

Cu (II) and Al(III) ions were chosen because there is a possibility that they will react with flavonoids *in vivo.* Copper is transition metal and essential trace element. As the important part of many cellular enzymes, copper can protect cells from oxidative damage. Aluminium is not essential trace element, but as the ingredient of the widely used antacid drugs, it could react with flavonoids *in vivo*, too.

## 2. Results and Discussion

### 2.1. Effects of flavonoids on the coagulation assays

Amongst the abundance of data related to pharmacological properties of flavonoids, experiments where Rut and Hesp were tested on the coagulation cascade have not been described yet. Coagulation consists of a series of zymogens that can be converted by limited proteolysis to active enzymes leading to the generation of thrombin, which in turn converts fibrinogen into fibrin. The effects of Rut and Hesp were investigated through three tests: aPTT, PT and TT. Activated partial thromboplastin time (aPTT) is a performance indicator measuring the efficacy of both the *intrinsic pathway* and the common coagulation pathways. It is used in conjunction with the prothrombin time (PT) which measures the *extrinsic pathway*. Thrombin time (TT), is a blood test which measures the time it takes for a clot to form in the plasma in which an excess of thrombin had been added.

Firstly, the effects of water solutions of different concentrations of Rut and Hesp on coagulation times were investigated, Table 1. Since flavonoids are barely soluble in water, the solutions with maximum possible overall concentration of Rut and Hesp were prepared, providing the maximum final concentration of 20 µM for Rut and 2 µM for Hesp in plasma. The effects of dilution were eliminated by adding the corresponding amount of water (Table 1).

The results from Table 1 showed that both Rut and Hesp at the investigated concentrations have no effects on either of the coagulation times. All values fall inside the standard deviation range. When comparing to the blank, no considerable difference in values was observed during incubation times up to 240 min.

Since it is not possible to increase the flavonoid concentration in water, we prepared ethanolic stock solutions with significantly higher concentrations of Rut (5.0 mM) and Hesp (2.5 mM). But being a solvent in the coagulation assays, ethanol generates its own effects. It is already known that ethanol denatures proteins by disrupting the side chain intramolecular hydrogen bonding forming new hydrogen bonds between the new alcohol molecule and the protein side chains. This may cause inactivation of coagulation factors and consequently, affect the coagulation cascade. Given that there is no data in literature about *in vitro* effects of ethanol in spiked plasma, we performed such experiments. Our results showed that ethanol alone changes all coagulation times, showing potent anticoagulant activity. By spiking plasma with different concentrations of ethanol and measuring the effects (data are not shown), a prolongation of aPTT, PT and TT, as well as extensive reduction of fibrin level were observed, all in a concentration-dependent manner. As a conclusion, we find it possible to use the ethanol beyond 20% v/v as the solvent. Thus, the plasma was spiked with ethanolic solutions of flavonoids to obtain final concentrations from 50 to 830 µM and 25 to 470 µM for Rut and Hesp, respectively, in 16.6% ethanolic solutions. Ethanol in the same amount was used as the vehicle control. The results are presented in Table 2.

The results showed that all coagulation times of plasma samples spiked with ethanolic solution of flavonoids were not significantly changed, compared to the control measurement. Only Rut in the highest concentration prolonged aPTT, but not in a statistically significant manner (p > 0.05), while TT and PT were unaffected. In order to evaluate whether the observed prolongation of aPTT was due to the effects on coagulation factors, the experiment with deficient plasma was performed for both flavonoids. The results are presented in Table 3.

Compared to the control measurement, some effects of Rut on factors VIII and IX can be observed, but the variations were not significant (p > 0.05). No effects on other coagulation factors were obtained, for either Rut or Hesp. In the literature, the binding of flavonoids to proteins has already been scrutinized and discussed. Bovine and human serum albumin, as well as insulin, were some of the target proteins [33,34,35]. It was found that flavonoid/protein interaction is not entirely hydrophobic in nature. Ionic interaction between the negatively charged OH groups of flavonoids and several ionic and polar amino acids at the putative binding site are involved in this molecular interaction. Thus, through the interaction of flavonoids with factors VIII and IX, the coagulation cascade could been triggered, what would lead to prolongation of aPTT. Since the literature data pointed out that flavonoids react with proteins in their ionisated form, the mol fraction of flavonoid in anionic form can be calculated through following equation [36]:

$$\Phi_{0}=\frac{1}{1+[H^{+}]K_{1}+{\left[H^{+}\right]}^{2}K_{1}K_{2}+\ldots .}$$

where K_1_ and K_2_ represent the first and second protonation constants of flavonoid. Knowing the protonation constants of Rut and Hesp [12,13,14,15,16,17], it is possible to calculate the active flavonoid concentrations in standard human plasma at physiological pH that actually may cause effects on coagulation pathway: approximately 9% of the overall Rut concentration (60µM) and 0.1% of the overall Hesp concentration (0.3 µM). Considerably higher mol fraction of anionic form of Rut than Hesp, may be the reason for aPTT prolongation, although these concentrations were well under the concentration of 1 mM of ATS and QTS which was reported to show significant prolongation of aPTT [30].

### 2.2. Effects of flavonoid-metal complexes on the coagulation assay

The pharmacological activity of flavonoids is believed to increase when they are coordinated with metal ions [18,19,20,21,22,23,24,25,26]. Thus, the primary goal of this survey was to investigate whether the effects of complexes on coagulation pathway were increased compared with mature flavonoids. Four complexes: Rut-Al, Rut-Cu, Hesp-Al and Hesp-Cu were investigated on the coagulation times and the results were presented in Table 4.

The concentrations of free flavonoids and metal salts were the same as in the complexes. All samples were prepared in 50% ethanol to provide final 14.3% ethanolic solution in plasma. Compared to the control measurement, Rut and Hesp in the concentration of 714 µM and 357 µM, respectively, showed no prolongation of aPTT, and no effect on TT and PT. Also, metal salts did not affect neither of coagulation times. Compared to free flavonoids, as well as the control, the prolongation of aPTT was unambiguous and significant for all complexes: Rut-Al and Hesp-Cu prolonged aPTT significantly (p < 0.001), Hesp-Al also prolonged aPTT significantly (p < 0.05), while Rut-Cu prolonged aPTT, but not in statistically significant manner (p > 0.05). Neither of complexes altered TT and PT, since all variations fall inside the range of standard deviation.

It was already emphasized that flavonoids bind to proteins in their dissociated form. During our longtime research, we found out that flavonoids also form complex compounds in their dissociated form [12,13,14,15,16,17]. Thus, if chelation of metal ions were the only process, aPTT would be in the same range as the control measurement, since the amount of free flavonoids had to be diminished. But all four complexes extend aPTT over control measurement, acting as anticoagulant agents.

Prolonged aPTT and unchanged PT and TT implies that factors from *intrinsic pathway* (VIII, IX, XI and XII) were only affected, what was confirmed in the test with deficient plasma, Table 3. The effects of Rut-Al and Hesp-Cu complexes were observed on all factors from *intrinsic pathway*, but prominent on factors VIII and IX.

The mechanism of anticoagulant action cannot be deduced from coagulation tests alone, but some parallels between the flavonoid-metal complex features and their anticoagulant actions can be drawn. It is already known that the investigated complexes were mononuclear (with one central ion) with one ligand (Rut-Cu and Hesp-Al) or two ligands (Rut-Al and Hesp-Cu). Complexation with flavonoids as unidentate ligands leads to the formation of complexes that contain protons in addition to the metal ion and ligand (so called protonated complexes), which tend to dissociate. In human plasma, which is slightly basic media, the complexes are dissociated and negatively charged. Although the structural requirements that associate complexes to the physiological glycosaminoglycans could not be fulfilled, complexes can also bind to proteins (coagulation factors) in their anionic form what may lead to a non-specific inhibition and aPTT prolongation.

If we compare the composition and stability of complexes with clotting time, the same correlation could be underlined. Hesp-Cu and Rut-Al, as more stable complexes, having stability constants log β = 11.53 and log β = 10.9, respectively, prolong aPTT more than less stable complexes Rut-Cu and Hesp-Al with lower stability constants, log β = 4.7 and log β = 3.7, respectively. Rut-Cu and Hesp-Al, as a mononuclear complexes with 1:2 metal to ligand stoichiometry, are bigger molecules with different microenviroment then other two complexes. These complexes, as negatively charged, may fulfill the sterical requirements for non-specific interaction within binding cavity of target coagulation factors.

## 3. Experimental

### 3.1. Reagents

Rutin and hesperidin were acquired from Fluka (Buchs, Switzerland). Absolute ethanol, Cu(NO_3_)_2,_ Al(NO_3_)_3,_ were obtained from Merck (Darmstadt, Germany). All reagents were of *p.a*. grade and were used without further purification. The standard stock solution of Rut and Hesp was prepared dissolving flavonoids in absolute ethanol to achieve concentration of 5.0 × 10^−3^ M and 2.5 × 0^−3^ M, respectively. The stock solutions of metal salts were prepared in water in the concentrations five times higher than the flavonoid concentration.

### 3.2. Complex preparation

All investigated complexes were 50% ethanolic solutions and were prepared in a 20 mL volumetric flasks, by mixing appropriate volumes of the standard stock solution of Rut or Hesp (V_1_), absolute ethanol (V_2_), water (V_3_) and stock solution of metal salt (V_4_) following this order. To obtain final solutions of the required molar concentrations in 50% (v/v) ethanol, it is necessary that V_1_ + V_2_ = 10 mL and V_3_ + V_4_ = 10 mL. Since volumes are not strictly additive, volumetric flasks were filled to the mark with 50% ethanol. A blank solution without Rut/Hesp was prepared in a 20 mL volumetric flasks, by mixing appropriate volumes of absolute ethanol, water and metal salt solution [12,13,14,15,16,17]. All solutions were stored in refrigerator, protected from daylight and appeared to be stable during the period of study.

### 3.3. Measurement of clotting assays

The activated partial thromboplastin time (aPTT), prothrombin time (PT) and thrombin time (TT), as well as coagulation factors assays, were carried out in a ACL 9000 coagulation analyzer (IL, Instrumentation Laboratory, Lexington, MA, USA) using standard human plasma provided by the same producer. Standard plasma (0.5 mL) was spiked with sample (0.1 mL of flavonoids or 0.2 mL of complexes) and the coagulation times were measured according to instructions provided by the producer. All reagents used were from IL company: Recombiplastin 2G^®^ (for PT measurement), APTT-SP liquid^®^ (for aPTT), Thrombin time^®^ (for TT) and Factor deficient plasma^®^ (for coagulation factors), added in the quantity defined by producer. For the determination of factors from *extrinsic pathway* (II, V, VII and X) plasma with sample was mixed with deficient plasma (missing the investigated factor) and thromboplastin Recombinant 2G^®^. For the determination of factors from *intrinsic pathway* (VIII, IX, XI and XII) plasma sample was mixed with deficient plasma and APTT-SP liquid^®^ reagents in the presence of 0.025 M CaCl_2_.

All values are given in seconds as the mean of six measurements ± SD. Differences between control and sample measurement were assessed by *Mann*–*Whitney* test. A value of P less than 0.05 (p < 0.05) was considered statistically significant. For coagulation factors assay, the transformation of seconds into percentages was done using standard diagrams made for every new lot of reagens.

## 4. Conclusions

Water solutions of Rut and Hesp at concentrations of 20 and 2 µM, respectively, showed no effects on *in vitro* plasma coagulation. Ethanolic solution of Rut at an overall concentration of 830 µM (which corresponds to a mol fraction of 60 µM of anionic Rut form), showed prolongation of aPTT due to interaction with factors VIII and IX. All investigated complexes prolonged only aPTT, (Rut-Al and Hesp-Cu significantly, p < 0.001) and had no effects on PT and TT. The effects of Ru-Al and Hesp-Cu complexes were observed on all factors from the *intrinsic pathway*. Rut-Cu and Hesp-Al, as stable, mononuclear complexes with 1:2 metal to ligand stoichiometry, may fulfill the steric requirements for non-specific interactions within binding cavity of target coagulation factors what results in anticoagulation effects. In summary, the observation that complexes are effective anticoagulant agents, suggests that further investigations of these flavonoid-metal complexes *in vitro* are merited in order to define the mechanism(s) of action.

## Figures and Tables

**Table 1:** The effect of different concentrations of water solutions of Rut and Hesp on coagulation times. The maximum possible concentration was c_Rut_ = 20 µM, c_Hesp_ = 2 µM.

	aPTT, s	TT, s	PT, s
Standard plasma	31.9±0.2	16.0 ± 0.3	9.5 ± 0.1
Control (plasma + water)	32.6 ± 0.9	17.5 ± 0.4	9.8 ± 0.3
**Rutin**, µM			
20	33.2 ± 0.7	18.2 ± 0.2	10.2 ± 0.4
9	30.7 ± 0.4	17.8 ± 0.9	10.9 ± 0.3
5	29.9 ± 1.1	16.9 ± 1.3	11.0 ± 0.5
2	30.5 ± 0.4	17.1 ± 0.5	10.8 ± 0.5
1	32.1 ± 0.3	18.0 ± 0.4	11.0 ± 0.6
0.6	32.8 ± 0.8	17.9 ± 0.6	11.8 ± 0.2
**Hesperidin**, µM			
2	31.7 ± 0.9	17.4 ± 0.5	10.0 ± 0.4
1	30.5 ± 0.6	16.8 ± 0.7	10.9 ± 0.3
0.5	32.0 ± 0.5	17.7 ± 0.9	10.5 ± 0.2
0.25	30.3 ± 0.5	16.9 ± 0.4	11.0 ± 0.5
0.12	32.1 ± 0.6	17.7 ± 0.8	11.7 ± 0.5
0.06	31.1 ± 0.8	20.0 ± 0.3	11.0 ± 0.6

**Table 2:** The effect of Rut and Hesp in 16.6% ethanolic solution on coagulation times. The maximum possible concentration of flavonoids: c_Rut_= 830 µM, c_Hesp_= 470 µM.

	aPTT, s	TT, s	PT, s
Standard plasma	29.9 ± 0.2	11.8 ± 0.1	10.9 ± 0.2
Control (plasma+ ethanol)	**49.9** ± 0.8	**19.8** ± 0.9	**16.7** ± 0.8
**Rutin**, µM			
830	55.9 ± 0.6	19.2 ± 0.8	16.1 ± 0.3
400	50.7 ± 0.6	19.1 ± 0.8	15.8 ± 0.9
200	49.1 ± 0.9	18.9 ± 0.5	15.9 ± 0.9
100	48.5 ± 0.3	18.1 ± 1.1	16.0 ± 0.8
50	49.2 ± 0.9	19.2 ± 0.9	16.2 ± 0.7
**Hesperidin**, µM			
470	51.8 ± 0.9	18.8 ± 0.8	16.6 ± 0.3
200	49.6 ± 0.9	19.8 ± 0.3	15.0 ± 1.1
100	49.4 ± 0.6	18.7 ± 0.4	16.2 ± 0.7
50	49.3 ± 0.7	18.9 ± 0.9	16.1 ± 0.5
25	47.1 ± 0.2	19.7 ± 0.8	15.0 ± 0.6

**Table 3:** The effect of Rut and Hesp and their complexes on the coagulation factors. The values were given in percentages.

Coagulation factors	II	V	VII	VIII	IX	X	XI	XII
Standard plasma	99.9	110.0	116.0	91.4	116.0	100.0	80.9	90.9
Control (plasma+ ethanol)	77.3	71.1	90.0	38.3	53.7	82.3	48.3	56.3
**Rutin**	79.5	66.4	87.8	29.2	45.0	84.0	44.8	54.9
**Hesperidin**	77.3	70.1	92.7	38.7	51.5	84.3	47.2	55.9
Rut-Al	73.3	59.1	88.9	24.1	30.7	83.0	40.1	49.3
Rut-Cu	75.8	70.1	92.4	38.1	50.6	82.1	49.3	50.5
Hesp-Al	71.3	70.0	88.2	29.9	35.9	82.3	41.5	50.6
Hesp-Cu	72.3	66.8	90.1	27.1	32.6	84.0	40.9	51.2

**Table 4:** The effects of flavonoid-metal complexes on coagulation times. The concentrations of flavonoids are the same as in the complexes: c_Rut_ = 714 µM, c_Hesp_ = 357 µM. All samples were prepared in 50% ethanol providing 14.3% ethanolic plasma solution.

	aPTT, s	TT, s	PT, s
Standard plasma	29.1 ± 0.2	18.0 ± 0.1	10.0 ± 0.2
Control (plasma+ ethanol)	**38.1** ± 0.4	**26.8** ± 0.7	**18.9** ± 0.4
Rutin	38.9 ± 0.2	26.8 ± 0.8	17.9 ± 0.4
Hesperidin	37.7 ± 0.5	27.0 ± 0.9	18.3 ± 0.5
Al(NO_3_)_3_	36.9 ± 0.4	26.9 ± 0.6	18.4 ± 0.9
Cu(NO_3_)_2_	37.0 ± 0.2	27.1 ± 0.2	18.0 ± 0.5
**Rut-Al**	**66.6** ± 0.9	**25.9** ± 0.4	**18.2** ± 0.5
**Rut-Cu**	**40.9** ± 0.4	**27.2** ± 0.5	**19.0** ± 0.4
**Hesp-Al**	**54.9** ± 0.5	**28.0** ± 0.9	**19.1** ± 0.7
**Hesp-Cu**	**63.7** ± 0.9	**27.7** ± 0.8	**18.5** ± 0.9
